# The care receivers perspective: How care‐dependent people struggle with accepting help from family members, friends and neighbours

**DOI:** 10.1111/hsc.12906

**Published:** 2019-12-09

**Authors:** Femmianne Bredewold, Loes Verplanke, Thomas Kampen, Evelien Tonkens, Jan Willem Duyvendak

**Affiliations:** ^1^ University of Humanistic Studies Utrecht The Netherlands; ^2^ University of Amsterdam Amsterdam The Netherlands

**Keywords:** care receivers, care reforms, community care, informal care, reciprocity

## Abstract

In many countries in north‐western Europe, the welfare state is changing, and governments expect a great deal of informal care. In the Netherlands, citizens are also increasingly expected to rely on informal instead of professional care. In this study, we aim to determine to what extent Dutch care‐dependent people want to rely on social network members and what reasons they raise for accepting or refusing informal care. To answer this question, we observed 65 so‐called ‘kitchen table talks’, in which social workers assess citizens’ care needs and examine to what extent relatives, friends and/or neighbours can provide help and care. We also interviewed 50 professionals and 30 people in need of care. Our findings show that a great deal of informal care is already given (in 46 out of 65 cases), especially between people who have a close emotional bond. For this reason, people in need of care often find it difficult to ask their family members, friends or neighbours for extra assistance. People are afraid to overburden their family members, friends or neighbours. Another reason people in need of care raise against informal care is that they feel ashamed of becoming dependent. Although the government wants to change the meaning of autonomy by emphasising that people are autonomous when they rely on social network members, people who grew up in the heyday of the welfare state feel embarrassed and ashamed when they are not able to reciprocate. Our findings imply that policymakers and social professionals need to reconsider the idea that resources of informal care are inexhaustible and that citizens can look after each other much more than they already do. It is important that social policymakers approach the codes and norms underlying social relations more cautiously because pressure on these relations can have negative effects.

**What is known about this topic**

- In the changing welfare state, the meaning of autonomy has changed. People are no longer considered independent when they rely on professional care, but when relying on their social network.
- Exchange in informal care relations manifests itself differently within various relationships.
- The closer the emotional bond, the less people talk about the exchange in the relationship.

**What this paper adds**

- Care receivers try to comply with the exchange codes that fit the specific relationship.
- Care receivers feel guilty and embarrassed when they need to receive more than they are able to give in return.
- Family members who give informal care risk becoming overburdened, particularly because they are not expected to discuss an imbalance in their relationship.

## 1 INTRODUCTION

In the Netherlands, the social domain has undergone a far‐reaching change. Citizens have to become more self‐reliant and face the new reality that the availability of professional care financed by the government is no longer self‐evident. Citizens are increasingly expected to assume more responsibility to find solutions for their care needs themselves and to look after each other as much as possible. This new policy paradigm is motivated by the conviction that the welfare state, developed since the 1960s, is no longer sustainable due to economic (several financial crises) and demographic (aging population) reasons (Grootegoed, Bröer, & Duyvendak, 2013).

The restructuring of the welfare state took place hand in hand with the decentralisation of social care and social assistance to the municipalities in 2015 (Fenger & Broekema, 2019). The transfer of these responsibilities was accompanied by significant budget cuts of about 15%–20% (Bredewold, Duyvendak, Kampen, Tonkens, & Verplanke, 2018:221). The Dutch government took some concrete measures in line with their new policy paradigm to realise these cuts. Several nursing homes were closed with the argumentation that old people can be best cared for in their home and by their social network, instead of moving to (much more expensive) residential care facilities. The government also made considerable cuts in day care and personal assistance for psychiatric patients, people with intellectual disabilities, and frail elderly, who live independently. Henceforth they have to rely as much as possible on their social networks (Fenger & Broekema, 2019). Social professionals are expected to fulfil an important role in this ‘activation policy’. It is their task to assess citizens’ care needs and to determine to what extent family and other social network members can provide help (Newman & Tonkens, 2011).

Because of the retrenchment of the welfare state in western countries and this ‘activation policy’, informal care has been subject of a great deal of research. Research on informal care focuses on the scope and extent of the tasks of informal caregivers (e.g. Lapierre & Keating, 2013), describes the burden and costs experienced by caregivers (e.g. Pearlin, Semple, & Skaff, 1990; Prevo et al., 2018), investigates the support that caregivers need to maintain their caring role (e.g. Zapart, Kenny, Hall, Servis, & Wiley, 2007), focuses on divisions of responsibilities between informal caregivers and professionals (e.g. Jacobs, Broese van Groenou, Boer, & Deeg, 2014; Wittenberg, Kwekkeboom, Staals, Verhoeff, & Boer, 2017) and provides insight into the intentions to give care from the caregivers’ perspective (e.g. Broese van Groenou & De Boer, 2016).

We found a few studies that concentrate on the changing welfare state and the higher expectations of informal care from the care receiver's perspective (e.g. Aronson, 2006; Grootegoed et al., 2013; McCann & Evans, 2002). These studies do not, however, give insight into how the type of relationship between people in need of care and possible informal carers influences the decision of the former to accept or refuse informal care. We think it is important to pay attention to this relationship, as it sheds light on the position of the care receiver and draws attention to dependency in relations (Tronto, 1993). Our study will concentrate on this care receiver's perspective, taking the anthropological and sociological gift theory as a starting point. This theoretical framework gives us insight into the interrelatedness between care giver and care receiver and can teach us more about what we might expect from the exchange in informal care relations in a shrinking welfare state. Before we proceed to our outcomes, we will describe this theoretical framework.

Research shows that the exchange of material goods or intellectual property is fundamental to contact between people (Komter, 1998, 2003; Mauss, 1990[1923]). In the market, between strangers, reciprocity exists through immediate equal exchange. In relationships between family members and close friends, immediate equal exchange is not necessary. Nevertheless, expectations of reciprocity also characterise these relationships. The principle of reciprocity assumes that giving is called upon by receiving, which puts into action a chain of giving‐receiving‐giving. This reciprocity is the start of a relationship as is depicted by Mauss, 1990[1923]. According to this research, we may conclude that reciprocity is a common norm and pattern in relationships between people and that this exchange (giving‐receiving‐reciprocating) manifests itself differently within various relationships.

Research shows that the weaker the emotional bond is, the more important is the balance of reciprocity, and the more demands are placed on time, quantity or quality of the probable gifts given in return (Komter, 1998, 2003; Sahlins, 1972). In the relationship between parents and children, for example, where the emotional bond is close, this balance is absent. Parents tend to serve their children hand and foot; they are at their children's disposal and do not expect their children to return this favour. However, in the relationship between, for example, neighbours, where the emotional bond is usually less close, reciprocity is the norm. Neighbours tend to exchange services based on a balanced exchange (Komter, 1998, 2003; Sahlins, 1972).

**Figure 1:**
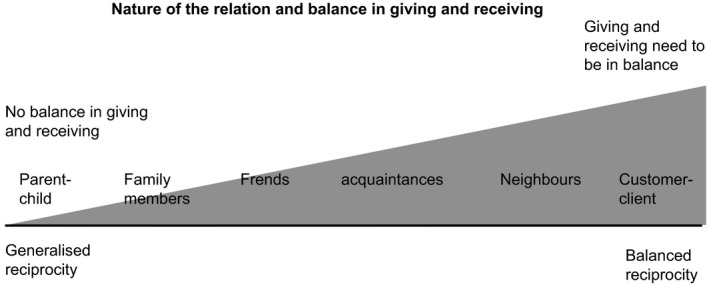
Reciprocity in relations

Sahlins (1972) introduced the term ‘generalised reciprocity’ for relationships where expectations to receive something in return are less specific, with no demands placed on the time, quantity or quality of the probable gifts given in return. Generalised reciprocity is a characteristic of people with a close emotional bond. Sahlins speaks of ‘balanced reciprocity’ when relationships are less personal and more direct, and equal exchanges are expected without delay. How the social emotional bond relates to the balance of reciprocity is pictured in Figure 1.

Various studies in western countries show that people try to live up to the norm of the specific relationship (Bredewold, Tonkens, & Trappenburg, 2016; Ekeh, 1974; Komter, 2007; Uehara, 1990, 1995). These studies indicate that people find it difficult to ask for help, regardless of how desperately they need it. People find it especially difficult when they think it does not fit their relationship with the caregiver. There is a high degree of ‘reluctance to ask’ (Linders, 2010), especially in relations where reciprocity is the norm. Not being able to reciprocate leads to feelings of shame and guilt (Grootegoed et al., 2013).

We are aware that this is a theoretical model based on research in western countries, and that the model will not suit all cultures. In fact, research convincingly shows that care perceptions and ideas about informal care vary from culture to culture (Cohen, Sabik, Cook, Azzoli, & Mendez‐Luck, 2019; Verbakel, 2018). Research among the largest group of people with a non‐western background in the Netherlands (Turkish, Moroccan and Surinamese Creole families) shows for example that ideas about care in migrant groups are more in agreement with the current Dutch regime of ‘active citizenship’ than the attitudes of citizens with a western background. Non‐western citizens have a stronger informal care attitude and are more sensitive to pressure to provide informal care (Van den Berg, 2014; Van Wezel et al., 2016).

In summary, anthropological and sociological theory and empirical research suggest that reciprocity is the basis for interpersonal relationships and that every relationship has its own balance of giving and receiving that people tend to live up to. It seems important to respect this balancing in relationships. In this article we will further examine what happens when social professionals try to intervene in relationships and how care receivers react to this pressure. We aim to answer the following question: *For what reasons do care‐dependent people accept or refuse informal care, and how is informal care intertwined with the relation between care giver and receiver?*

## 2 METHODS

### 2.1 Observation during ‘kitchen table talks’

As part of the Dutch care reforms, the local government is now responsible for people in need of care. These people have to ask their local government for support. In most Dutch municipalities so‐called *social district teams* are the first and most important point of contact for residents who require support. Social workers from these teams visit care‐dependent residents at their homes and discuss their needs (these conversations are called ‘kitchen table talks’). Social workers assess the need for care and examine to what extent family members, friends or neighbours can provide help and whether professional care has to be provided. As people in need of care are no longer automatically entitled to professional care since the Dutch care reforms in 2015, they are dependent on how the social worker in their specific municipality assesses their request for help.

In line with the government's policy to live as long as possible independently in the community, professionals of the social district teams have to encourage their clients’ network to undertake various tasks which are important to keep a household running, for example do the shopping, clean up, take on administrative tasks, accompany visits to the hospital or offer emotional support. As in the Netherlands physical and medical support such as help with showering and administer medication is regulated by another law, social professionals who do undertake the kitchen table talks do not need to insist on such forms of support by the client's social network.

Between January 2015 and July 2017, three researchers observed and analysed 65 of these kitchen table talks to determine how care‐dependent people react to the new incitement to ask their social network for help and care. Our research was conducted in six Dutch cities. Next to the citizen with care needs, a social worker was present at the kitchen table talk and sometimes a family member or friend to give some support and help to express the needs of the person needing care. This support by a family member or a friend turned out to be especially helpful when people had difficulties expressing themselves clearly for example in case of people with an intellectual disability or people with dementia. The social workers asked their clients in advance if we might join the kitchen table talk as an observer. The professionals and citizens were informed about the research and gave their consent. We aimed for a sample of residents of different ages, impairments and care needs, but as we were dependent on (a) the efforts of the social workers to approach clients and (b) the approval of the clients themselves, the sample is not in all respects a reflection of the entire population. Table 1 shows the background characteristics of the residents in our sample.

**Table 1:** Background characteristics of people in need of care

	*N *= 65	%
Gender		
Male	23	35
Female	42	65
Age		
0–20	1	2
20–60	32	49
60<	32	49
Primary reason for needing support^a^		
Intellectual disability	4	6.5
Psychiatric or psychosocial needs	18	26
Dementia or other memory problems	4	6.5
Temporary physical constraints	5	7.5
Prolonged physical constraints (including old age)	31	46
Overburdened informal carers	5	7.5

a More answers possible.

During the kitchen table talks, we observed the setting, interaction, actions of the professional, and reactions of the client and other attendees (Patton, 2002). We registered our observations on three levels. First, direct field notes were taken on the spot. Second, a comprehensive report of the kitchen table talk was written immediately afterwards. Third, the researcher kept research diaries in which he or she noted general impressions and thoughts on the various observed kitchen table talks as well as reflections on the research process. During the research process, the research team regularly shared and discussed their findings.

### 2.2 In‐depth interviews

To gain insight into the different ideas and experiences concerning the new policy paradigm of self‐reliance, we interviewed care‐dependent citizens and social workers. After observing the kitchen table talks, we asked them if we could interview them separately. Thirty residents and fifty professionals agreed. With the residents, we discussed their ideas and opinions on the new government policy. We talked about their care needs, what they think about asking their family members, friends or neighbours for help and who they dare to ask for what kind of help and support. Social professionals were asked about their opinions and experiences on stimulating citizens’ self‐reliance and informal care by the social network. We spoke about possibilities and barriers they were facing when stimulating care‐dependent citizens to ask their relatives for support. The interviews relied on a topic list. All interviews were audio‐recorded and all respondents gave informed consent.

### 2.3 Data analysis

The interviews were transcribed verbatim, and the observations were reported in detailed logbooks. The researchers who conducted the fieldwork (three of the five authors) analysed the transcripts of the interviews and the logbooks of the observations using a coding scheme (code tree) based on a literature study (reasons to accept or hesitate help and support in informal care relations; generalised and balanced reciprocity). The researchers analysed the data after which they compared the coded data and decided on a final coding. For the analysis, we used the qualitative data analysis program ATLAS.ti. All data were anonymised. In the following section, we will describe the reasons people in need of care raise to accept or refuse informal care.

## 3 FINDINGS

### 3.1 Close emotional social bonds – naturally to count on but a risk of overburdening

Based on 65 observations of kitchen table talks, we can conclude that social network members already provided a great deal of informal care. In two‐thirds of the cases, family members, friends or neighbours were already involved. The greatest part of informal care is given by family, and female family members provide most of this care. Other studies also show that particularly family members provide help (Ekeh, 1974; Komter & Vollebergh, 2002; Uehara, 1995) and that informal care is mostly done by women (e.g. De Klerk, 2017). However, studies also show that women are the greatest recipients of care, which relates to the longer life expectancy for women than men (e.g. McGuire, Anderson, Talley, & Crews, 2007). The fact that women receive more care than men is also visible in our study (see Table 1) and here too we find an explanation in age. In the kitchen table talks, we saw many single elderly women in need of care.

In some cases, it was questionable whether more can be expected from the social networks. People in need of care explained that they are reluctant to ask their family members to provide more care because these family members do so much already. The care receivers are afraid to overburden their relatives. Although some research is showing a gender difference when it comes to the struggle with dependency—Roe, Whattam, Young, and Dimond (2001) found for example that especially women view receiving help as a loss of independence and as invasion of privacy—we found that women as well as men struggle with becoming dependent:

Man: I just don’t want to be dependent. (...) My sister is taking me to the doctor tomorrow. (He starts to cry). I don’t want to burden people too much. P192‐O

Moreover, precisely because the need to take care of family members seems to be self‐evident, we also witnessed distressing situations and overburdened family members. Family members give without asking something in return and don't want to talk about a balance in the relationship. For example, during one of our observations, we witnessed a man who had been caring for his paralysed wife for a long time:

Man: (sighing) It's quite something, but I really can't bring her to a nursing home!

Professional: Is it difficult to take care for her?

Man: I'm having a tough time, I can hardly cope, I have COPD, asthma, rheumatism [...].

Professional: You told me you don’t want to ‘get rid of’ your wife. You want to keep her at home as long as possible.

Man (sobbing): Yes, that's why I still try to manage. P49‐O.

Other research also makes clear that much informal care is given and that informal carers feel regularly (over)burdened and struggle with managing their own household (e.g. De Klerk et al., 2017; Mello et al., 2017; Peetoom, Lexis, Joore, Dirksen, & Witte, 2016: 93–94). Because of these distressing situations, care receivers do not want to increase the burden of family members and tell professionals that the capacity of their social network members is limited and reached.

### 3.2 Protection of the nature of a relationship—family relations under pressure

We found that there are more reasons why people don't want to ask their family for (more) help. In some cases, people are afraid that their request for help will change the relationship. By not asking, people try to protect the nature of their relationship. Our study shows that young people and adolescents in particular want to disengage from their parents as a natural process of self‐reliance. That is why these young people reject their parents’ assistance. A quote from a kitchen table talk of a woman with psychiatric disabilities in her early twenties:

Social worker: Well and about your social network?

Woman: Honestly, I don’t expect anything from people in my network. My sister, my father and my mother treat me like a little girl, but maybe I'm stronger than they are. I’m very thoughtful, and I think I understand people quite well, but they treat me like a baby. They always say: “No, you can’t do that, and you shouldn’t do that.” P110‐O

Vice versa, we saw in our research elderly people who don't want to become dependent on their children. Raised in the heyday of the welfare state, elderly people are used to professional care financed by the state. Not being dependent on their social network made elderly people all those years feel autonomous. Now that circumstances have changed, and the all‐caring welfare state is no longer self‐evident, elderly people are more or less forced to rely on their children for care, and this situation can make them feel embarrassed:

Care dependent elder woman: The situation is just very awkward. I'm dependent on my daughter now. That's not how it’s meant to be. P63‐O

During a kitchen table talk, a woman with physical problems explained why she does not want to ask her daughter to take her to the hospital:

Social worker: So, am I right when I conclude that you want to maintain a good relationship with your daughter, and that you therefore do not ask her to accompany you when you need to visit the hospital?

Woman: Yes, I am too independent for that. Then, I'd rather do something nice with her. That is also why I appeal to the municipality, that they help me. P209‐O

Even when the children themselves unmistakably explain that they want to help their mother, it is still very difficult for the mother to accept it:

Daughter 1: For my mother, it's so difficult to place such a burden on us, but that's the way she feels. We both work full time, but for us it's not a problem.

Daughter 2: No, it really doesn't feel like a burden. P164‐O

Elderly people who need physical care also try to safeguard the nature of their relationship with their spouse or children against intimate acts. In their opinion toileting, bathing and showering are too intimate; they do not want to bother their spouse or children with these activities. These findings correspond to other research (e.g. Szebehely & Trydegard, 2012: 306; Roe et al., 2001). This is a fragment from a kitchen table talk in which we find struggles with intimate care:

Woman: I ‘m no longer able to go to the toilet. I have a potty now. But my husband doesn't like me going on the potty, he doesn't like emptying it, he thinks it's dirty. So now I just don't go, but that hurts. At one point, I couldn't pee anymore. P204‐O

These different examples make it clear that, especially for elder people, it is easier said than done to rely on their social network for help instead of turning to the government for public‐funded care. To rely on social network members is in their experience contrary to the ideal image of the autonomous citizen and, therefore, very difficult to practice. While the government tries to change the ideas of autonomy by arguing that being dependent on your social network is now preferable to being dependent on professional care, we found that people in need of care did not so easily adjust to this new paradigm. They have other norms in mind when interacting with their family members: do not burden the family members too much and act as autonomously as possible.

### 3.3 Friendship under pressure

Care‐dependent people not only think it is difficult to ask family members for help; we found they also find it difficult to ask this from friends. Equality and balance in giving and receiving are an important basis for friendship according to the gift exchange theory, and care‐dependent people are no exception to this. They see it as a threat to the friendship when they ask their friends for care or help. A man with psychiatric problems tells:

*And friends, yes, I do not want to bother them too much with my situation. Because, I really feel ashamed. Look, I'm 38. I don’t have a job. I live in a flat in one of the worst neighbourhoods of this city. I am depending on care and must be taken by the hand. That is unworthy of a man.* P4‐CI

A young woman with psychiatric problems explains why she does not want to burden her friends:

*I mainly have contacts and friends outside of the world of psychiatry, and I find that extremely valuable. But you have to be very careful that you keep a proper balance in what you ask of these people; they must not take care for you. It is rather difficult for people to maintain friendship with someone like me. If it works well, that is quite an achievement. You want to take care of each other, but you do not want to take over the role of care provider because then it is no longer an equal friendship.* P11‐CI

### 3.4 Neighbourly contacts under pressure

Professionals also asked their clients if they can rely on support from their neighbours. However, most of them agreed with (western) unspoken neighbourly rules that contact with neighbours is supposed to be light and superficial:

Woman: We’ve always been lucky to have nice neighbours. They’ve lived here for a long time now, and we always clean each other’s driveway when it snows.

Social worker: So, you can ask your neighbours for support?

Woman: Oh no, we wouldn’t ask them for support. P98‐O

Other research shows that neighbours prefer not to assist in daily care because they do not want to interfere in each other's private domains. Neighbours maintain distance to prevent inconvenience and neighbourly disputes (Bulmer, 1986, 1987; Jacobs, 1960; Linders, 2010).

### 3.5 Self‐reliance in practice

Apparently, many people in need of care do not want to draw on their social network members because they do so already and they want to protect the nature of their relationships. Asking network members to provide more care or assistance seems to put pressure on their relationship, which they want to avoid. People cherish their relationships and do not want to risk a disturbance of the existing ‘balance’. Professionals sometimes try to extend the boundaries by asking whether clients can ask for slightly more help and support, but when they explain why they do not want to ask their network members for more help, professionals accept this:

Social worker: The initiative must come from people themselves. It’s all right if we focus more than we used to do on relying on family members, friends and neighbours, but it’s a fine balance. You can’t just say, “Your father has to do these things for you.” We don’t want to work that way. You might damage the relationship if your pressure on help and care is beyond the scope of the relationship. P14‐PI

Table 2 shows how often professionals mapped the social network and how many times they asked members to help. In only 3 out of 65 cases professionals did succeed in involving the social network. In 15 cases, the professionals tried to involve the social network but without success, and this outcome was related to the reasons explained above. In 45 cases, the social network was mapped, but social workers did not ask if the network could help because clients had already mentioned during the conversation that the network gave a great deal of help, or informal care was not (yet) necessary (12 cases).

**Table 2:** Involvement of social network after kitchen table talk

Number of times social professionals involved social network	*N* = 65
Network involvement successful	3
Spoken about involvement network, but not successful	15
Network mapped, but not asked for help	45
Network not mentioned	2

Instead of stimulating self‐reliance as prescribed by the Dutch government, professionals concluded that many people needed professional care. We found out professionals recommended professional care in approximately 53 of 65 cases.

## 4 DISCUSSION AND CONCLUSION

This study contains some limitations. First, the current study draws on a small sample of 65 observations, 30 interviews with people in need of care and 50 professionals. Thus, firm conclusions cannot be drawn from this study. Second, we only conducted research in cities. Informal care may certainly be different in villages (McKenzie, McLaughlin, Dobson, & Byles, 2010). Third, as most of our data were collected during kitchen table talks, where people had to negotiate with social workers whether they would receive professional or informal care, it seems plausible that reasons to avoid informal care were given more attention than reasons to accept it. Fourth, in our study we didn't interview (possible) care givers, so we were not able to check how they evaluate the relationship and whether more help and support was possible and would fit the relationship with the person in need of care. It seems very valuable to engage in future research possible care givers and compare their ideas with care receivers. Fifth, through our observations of the kitchen table talks, we were able to collect various background characteristics of respondents, but unfortunately religion and cultural background were no topic in these talks. Subsequently, we also did not touch upon it in the interviews with care receivers. We recognise that opinions about giving and receiving care are intertwined with religious and cultural differences, as is also shown in other studies (e.g. Cohen et al., 2019; Van Wezel et al., 2016; Verbakel, 2018), but we cannot draw conclusions how these variables are interrelated in this study.

However, our general findings, in particular the conclusion that every relationship has its own balance of giving and receiving which people tend to live up to, concur with previous research. This agreement makes us fairly optimistic regarding the validity of our main findings. Second, the triangulation of data allowed us to compare the insights from various perspectives. Moreover, it not only gave us insight into the opinions and thoughts of professionals and care receivers but also provided a glimpse of the lived practice of the current informal care policy in the Netherlands.

Based on our research, we conclude that the emphasis on self‐reliance is a great change compared to the dominant policy of the welfare state in the last decades of the 20th century. When the welfare state began to flourish in the 1960s, it was seen as a major step forward that people could appeal to public‐funded professional care instead of being dependent on their family. Nowadays, it is the other way around; people have to rely on their social network for help in the first place. The meaning of autonomy has changed; one no longer becomes independent by relying on the government but by relying on one's private social network. In the kitchen table talks and interviews with clients, we saw that this paradigm change is substantial and that people in need of care have difficulties internalising this new idea of autonomy. These people mainly grew up in the welfare state and still want to act as autonomous people who do not depend on their social network members.

For the time being, this new ideal of autonomy seems to have no influence on how people deal with the gift exchange in their specific relation. People still try to balance their gift exchange and do their best to comply with the exchange‐codes that fit the specific relationship. They are not yet ready to accept more ‘gifts’ and still struggle with their dependency.

It is important to take the age‐old theory of gift exchange (Mauss, 1990[1923]]) more seriously with regard to the recent informal care policy. This theory teaches us that family members do not expect something in return and give without asking. In this respect, it does not come as a surprise that we found so many caring family members. Nor is it surprising that we found so many overloaded family members. Overburdening in family relationships is a real risk, particularly due to the nature of these relationships. The boundless moral call of the government for citizens to care for each other and the corresponding change of the meaning of autonomy put even more pressure on these relationships. This pressure leads to exhaustion and overburdening of family relationships.

As women still perform a larger share of informal care tasks than men and also receive more care, a moral appeal on informal care seems to affect women the most. It is important that more attention is paid to this issue as it is obvious that gender inequality will increase as a result of the current government policy.

In addition, we conclude it is also important to take the gift exchange theory into account in regard to expectations of informal care by friends and neighbours. We found that care‐dependent people find it difficult to ask their social network for (extra) assistance because they value a certain balance between receiving and giving. Care‐dependent people feel ashamed and sometimes even guilty when they are unable to reciprocate. They do not want to build up ‘a debt’ in the relationship. We saw a high degree of reluctance to ask, especially in relationships where reciprocity is the norm. This finding also shows that we cannot see care recipients as only passive and dependent, as was also noted by Lambotte et al. (2018).

Our findings imply that the government needs to consider the limited capacity of social networks and the nature and complexities of social relationships when designing policy plans for informal care. While policymakers expect people to care for each other even more, our research shows that social professionals came across barriers related to social norms and codes underlying social networks. It seems important that policymakers and professionals take those codes and rules more seriously when developing and implementing informal care policy and that they bear in mind that the capacity of social networks is limited even as the capacity of women who provide the greatest deal of informal care.
